# Pyrroloquinoline-Quinone Is More Than an Antioxidant: A Vitamin-like Accessory Factor Important in Health and Disease Prevention

**DOI:** 10.3390/biom11101441

**Published:** 2021-09-30

**Authors:** Karen R. Jonscher, Winyoo Chowanadisai, Robert B. Rucker

**Affiliations:** 1 Department of Biochemistry and Molecular Biology, College of Medicine, University of Oklahoma Health Sciences Center, Oklahoma City, OK 73104, USA; karen-jonscher@ouhsc.edu; 2 Department of Nutrition, Oklahoma State University, Stillwater, OK 74078, USA; winyoo.chowanadisai@okstate.edu; 3 Department of Nutrition, University of California, Davis, CA 95616, USA

**Keywords:** pyrroloquinoline quinone, PQQ, cell signaling, nutrition, vitamins, inflammation, antioxidant

## Abstract

Pyrroloquinoline quinone (PQQ) is associated with biological processes such as mitochondriogenesis, reproduction, growth, and aging. In addition, PQQ attenuates clinically relevant dysfunctions (e.g., those associated with ischemia, inflammation and lipotoxicity). PQQ is novel among biofactors that are not currently accepted as vitamins or conditional vitamins. For example, the absence of PQQ in diets produces a response like a vitamin-related deficiency with recovery upon PQQ repletion in a dose-dependent manner. Moreover, potential health benefits, such as improved metabolic flexibility and immuno-and neuroprotection, are associated with PQQ supplementation. Here, we address PQQ’s role as an enzymatic cofactor or accessory factor and highlight mechanisms underlying PQQ’s actions. We review both large scale and targeted datasets demonstrating that a neonatal or perinatal PQQ deficiency reduces mitochondria content and mitochondrial-related gene expression. Data are reviewed that suggest PQQ’s modulation of lactate acid and perhaps other dehydrogenases enhance NAD+-dependent sirtuin activity, along with the sirtuin targets, such as PGC-1α, NRF-1, NRF-2 and TFAM; thus, mediating mitochondrial functions. Taken together, current observations suggest vitamin-like PQQ has strong potential as a potent therapeutic nutraceutical.

## 1. Introduction

Pyrroloquinoline quinone (PQQ) was first reported as a cofactor for bacterial dehydrogenases in the late 1960s [1]. PQQ’s recognition as a cofactor was important because, at the time, only nicotinamide cofactors and flavins were considered cofactors for bacterial dehydrogenases. Over the next fifteen years, the structure of PQQ was fully elucidated [2,3,4,5,6,7,8]. As a result, PQQ is now appreciated as part of a family of quinone cofactors, classified as quinoproteins, that are utilized by dehydrogenases and oxidases. The other quinoprotein cofactors include 2,4,5–trihydroxyphenylalanine quinone or topaquinone (TPQ), lysine tyrosylquinone (LTQ), cysteine tryptophylquinone (CTQ) and tryptophan phyloquinone (TTQ) [7,8].

Through nutritive and environmental exposures, PQQ affects essential biological processes, influencing mitochondriogenesis, reproduction, growth and aging. Multiple lines of evidence, in organisms ranging from fungi to mammals, indicate that the absence of PQQ from nutrient sources produces a broad assortment of abnormalities. Moreover, as a nutraceutical, PQQ attenuates clinically relevant conditions such as ischemia, inflammation, and lipotoxicity, and also has nootropic properties [9,10]. In this regard, genes essential for fatty acid metabolism and mitochondrial function are particularly targeted by PQQ.

Throughout this review, PQQ’s role in cell signaling will be a focus. PQQ serves as a catalytic accessory factor for lactate and other dehydrogenases in the oxidation of NADH to NAD+ which has emerged as a significant finding [11]. PQQ enhances NAD+-dependent sirtuin activity and the expression of sirtuins targets, such as PGC–1α, NRF–1 and 2, and TFAM [10]. Notably, few bioactive food components reduce the levels of reactive oxidative species as efficiently as PQQ [9].

## 2. Chemistry and Biologic Mechanisms of Action

### 2.1. General Properties

Pyrroloquinoline quinone (PQQ; 4,5-dihydro-4,5-dioxo-1*H*-pyrrolo [2,3-f]quinoline-2,7,9-tricarboxylic acid) is an aromatic water-soluble quinone whose chemical properties are analogous to combining the chemical features of ascorbic acid, riboflavin and pyridoxal-5-phosphate into one molecule. For example, both the oxidized (PQQ_ox)_ and reduced (PQQH_2_) forms of PQQ carry out redox cycling. PQQ also serves as a dehydrogenase cofactor, a free radical scavenger, and an amine oxidase catalyst [12,13]. PQQ is highly electrophilic and reacts with many substances (e.g., various carbonyl reagents, such as hydrazine, hydroxylamine and semicarbazides). Moreover, PQQ forms complexes with acetone, aminoguanidine, urea, various diamines, and divalent metals [12,13]. In addition, PQQ reacts with ammonia to produce an iminoquinone, and under acidic conditions, internal lactones are formed, in contrast to the formation of dihydrates under neutral and basic conditions [14].

**Figure 1:**
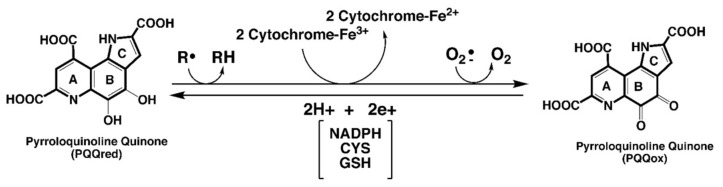
**PQQ and electron transfer reactions.** PQQ, in its reduced state, efficiently catalyzes electron transfer reactions. Potential substrates range from organic radicals to radical forms of oxygen. For many of the PQQ—dependent dehydrogenase systems, electron transfer may involve a heme-associated component. PQQ is reduced in reactions, utilizing either NAD[P]H, thiols such as cysteine and glutathione, enediols such as ascorbic acid, or aroxyl radical reductants such as the tocopherols.

PQQ does not easily self-oxidize or condense into inactive forms. Accordingly, when compared on a molar basis, PQQ can be 100 to 1000 times more efficient in redox cycling assays than other enediols, such as ascorbic acid and menadione, as well as many isoflavonoids, phytoalexins and polyphenolic compounds [12]. As a redox cofactor, PQQ is capable of catalyzing continuous redox reactions involving oxidation of thiols [12], riboflavin [12], ubiquinone, terminal cytochromes [15], α–tocopheroxyl radicals [16] and nicotinamide adenine dinucleotide cofactors [17]. As an antioxidant, PQQH_2_ can act as an aroxyl radical scavenger, even more so than α-tocopherol, against peroxyl radicals [16]. Moreover, in combination with α-tocopherol, PQQH_2_ can protect α-tocopherol by reducing α-tocopheroxyl radicals generated in the oxidation of polyunsaturated lipids. Figure 1 summarizes relationships seminal to PQQ’s role in redox processes and as an antioxidant.

Oxidized PQQ does not exert appreciable antioxidant activity in the presence of metals like copper; however, PQQox can catalyze the non-enzymatic oxidation of the epsilon amine function of lysyl groups to aldehydic functions in proteins, such as elastin and collagen, and the oxidation of pyridoxamine and pyridoxamine-5-phosphate to pyridoxal and pyridoxal-5-phosphate [12].


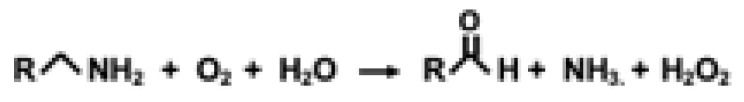


### 2.2. PQQ as an Enzyme Cofactor

Of the bacterial enzymes for which PQQ serves as a cofactor, most are glucose or alcohol dehydrogenases. The one exception is lupinine hydroxylase, from the genus *Pseudomonas* [13]. This PQQ—dependent quinoenzyme serves as an amine dehydrogenase, with the quinolizidine alkaloid from lupin plants, lupinine, as the substrate. Mechanisms involving hydride ion transfers are proposed for the oxidation of glucose and alcohol substrates. Metals ions, such as K^+^, Mg^2+^, Li^+^ or Ca^2+^ also serve catalytic roles [13].

**Figure 2:**
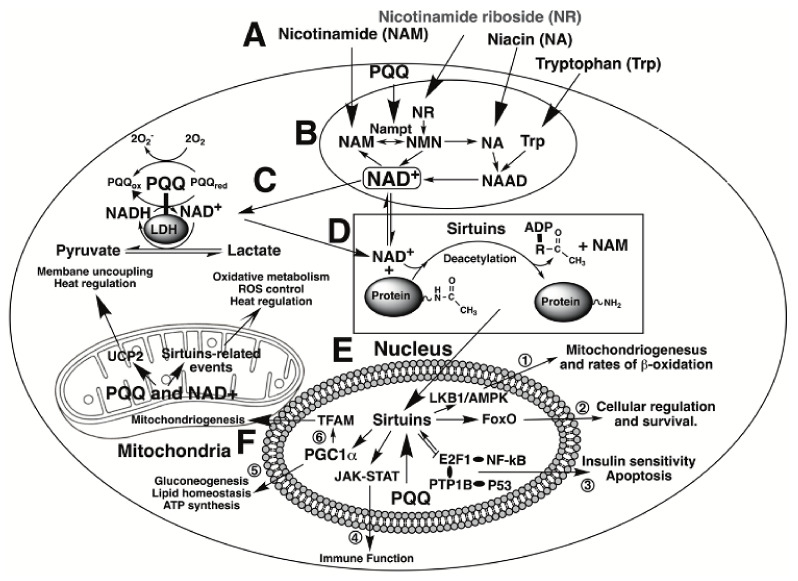
**PQQ and cellular NAD**^+^. Events elicited by PQQ and NAD+ are highlighted. At the top of the figure, **A**, niacin-related serum components are depicted that are important in the production of NAD+. Each interacts to generate NAD+ as indicated in the section encircled as **B**. PQQ plays a role in the process by enhancing the expression of nicotinamide phosphoribosyltransferase (designated as Nampt}. Increasing Nampt activity increases NAD+ cellular levels. NAD+ performs two principal functions; first, as a cofactor for dehydrogenases and reductases, such as lactic acid dehydrogenase (LDH), **C,** and second, as a co-substrate for sirtuin-catalyzed protein deacetylations as noted in **D**. Likewise, PQQ is a catalytic cofactor for LDH, C. PQQ facilitates the conversion of lactate to pyruvate. In the nucleus, **E**, NAD+, as a co-substrate for sirtuins, promotes targeted acetylation or deacetylation of proteins involved in cell signaling. Six examples are enumerated: (1) the LKBl/AMP-Kinase-pathway, important in the regulation of rnitochondriogenesis and rates of P-oxidation, (2) Forkhead box 0 transcription factors (FoxO) important to cellular proliferation and survival, (3) transcription factors involving NF-KB and P53 proteins, which regulate multiple aspects of innate and adaptive immune responsiveness, (4) the Janus kinase-signal transducer and activator of transcription JAK-STAT) pathway essential for processes related to hematopoiesis, lactation, and immune responsiveness, (5) peroxisome proliferator-activated receptor-gamma coactivator (PGC-lα), which plays a central role in cellular metabolism and ATP production and (6) the regulation of the mitochondrial transcription factor (TFAM) and other factors essential for mitochondrial genome replication. Finally, in the mitochondria, **F**. PQQ and NAD+ profoundly impact oxidative metabolism, ROS control, and heat regulation through events controlled by mitochondrial sirtuins and uncoupling proteins, such as UCP2.

Of fundamental importance, Akagawa and coworkers [18,19] have reported that PQQ serves a functional role in mammalian dehydrogenases such as lactate dehydrogenase (LDH). PQQ bound to LDH enhances NADH oxidation to NAD+ and pyruvate production [18,19]. Optimizing oxidative metabolism is inextricably linked with improved antioxidant defense and enhanced immune function. In this regard, NAD+ is a substrate for the deacylation reactions catalyzed by sirtuins, particularly sirtuin 1 and 3 (SIRT1 and SIRT3). The sirtuins are a family of signaling proteins essential to metabolic regulation related to oxidative processes. Some of the relationships are outlined in Figure 2.

To elaborate further, SIRT1 is primarily found in the nucleus and lesser amounts in the cytoplasm [11,20], whereas SIRT3 is generally localized in the mitochondria and associated with mitochondrial DNA transcription [11,21]. Peroxisome proliferator-activated receptor-gamma coactivator (PGC)-lα is one of the most critical factors controlled by sirtuin activity and is activated through deacetylation by SIRT1. PGC-lα acts as a transcriptional coactivator by enabling the assembly of other transcriptional enhancers, such as nuclear respiratory factors 1 and 2 (NRF-1 and NRF-2). Such factors are necessary elements in the cell required for maintaining energy homeostasis and engaging antioxidant response genes [22]. Moreover, Tchaparian et al. [23] have reported on the identification of other transcriptional networks responding to dietary PQQ supplementation. Among these are the 5′-adenosine monophosphate-activated protein kinase (AMPK), mitogen-activated protein kinase (MAPK), and the Janus kinase signal transducer and activator of transcription JAK-STAT) pathways, which aid in the control of cellular proliferation, mitosis, apoptosis and most aspects of the immune response [24,25,26,27,28,29,30,31,32].

## 3. PQQ’s Role in Prokaryotes and Fungi

### 3.1. PQQ Synthesis

Over one hundred prokaryotes have been identified as capable of PQQ synthesis and use PQQ as an enzymatic cofactor [33]. Most are Gram—negative and range in function from plant symbionts (e.g., root-associated rhizobacteria) to insect pathogens. The PQQ-requiring alcohol and glucose dehydrogenases in bacteria catalyze the oxidation of a wide variety of primary and secondary alcohols as well as catabolism of acyclic terpenes [13]. The glucose dehydrogenases (GDHs) exist in both membrane-bound and soluble forms [34,35]. Their DNA-derived protein sequences, however, are dissimilar, and there is little to no immunological cross-reactivity. Most attention has been given to the membrane bound GDHs localized to the periplasmic surface of cytoplasmic membranes [35], which are essential to bacterial respiration, oxidative metabolism, and growth. Moreover, their regulation is sensitive to changes in oxygen concentration and factors linked to cell growth. It is also important to note that some organisms that do not make PQQ will utilize it when available. A good example is the enteric bacterium *Escherichia coli* (*E. coli*), which has a membrane-bound PQQ-dependent GOH and an NADH-dependent GDH. The PQQ-dependent GDH is used preferentially when PQQ is available [13].

**Figure 3:**
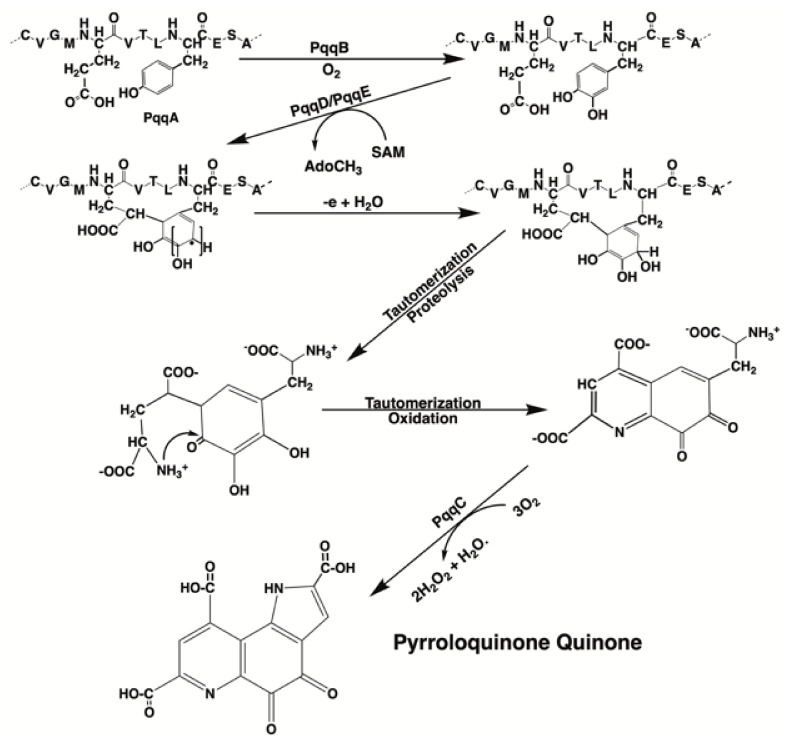
**PQQ synthesis.** The scheme shown for PQQ synthesis was adapted from the PQQ biosynthetic pathway in *Methylobacterium extorquens*. Multiple gene products (designated PqqA, PqqB, PqqC, etc.) are required for PQQ synthesis. PQQ synthesis is unusual in that PQQ is derived from the enzymatic annulation of peptidyl tyrosine and glutamic acid found in PqqA. The annulation of the glutamyl and tyrosyl residues is catalyzed by PqqD and PqqE. PqqD is a novel peptide chaperone that forms a ternary complex with the radical S-adenosylmethionine-requiring protein, Pqq E. The annulation step is then followed by oxidation, tautomerization, and the eventual proteolytic release of PQQ [36].

For those bacteria that make PQQ, five protein gene products, designated PqqA, PqqB, PqqC, PqqD, and PqqE, are required for PQQ production (Figure 3). Three gene products in the *pqq* operon, PqqB, PqqC, and PqqE, are members of large protein superfamilies. The roles for each gene product have been described by Zhu and Klinman [36]. Following synthesis, PQQ next binds to quinoenzymes, such as the bacterial glucose and alcohol dehydrogenases, at binding domains with the properties of β-propeller sequences. β-propeller sequences are characterized by 4 to 8 highly symmetrical blade-shaped beta-sheets arranged toroidally around a central axis. β-Propellers are found in all kingdoms of life. They function mainly as scaffolds for macromolecular interactions and ligand binding at catalytic sites [37].

### 3.2. PQQ and Fungi

The first to demonstrate a PQQ-containing enzyme in a eukaryote were Taketa, Matsumura, and coworkers [38,39,40,41], who isolated and characterized a novel PQQ-dependent sugar oxidoreductase from the basidiomycete, *Coprinopsis cinerea* (Cc). Basidiomycota are model organisms for studying fungal sex and mating types, mushroom development, and processes essential to multicellularity. The enzyme, an extracellular PQQ-dependent sugar dehydrogenase (CcSDH), comprises a signaling domain for extracellular secretion and domains for cellulose adsorption, PQQ binding, and cytochrome binding. Subsequently, Varnai and coworkers provided similar observations [15]. Although there are reports that yeast contains PQQ no specific PQQ-containing enzymes have yet been identified [42]. However, as Matsumura et al. [38] have emphasized, BLAST searches indicate the existence of many genes encoding homologous proteins to CcSDH in bacteria, archaea, amoebozoa, and fungi. Furthermore, phylogenetic analysis suggests those identified as quinoproteins are widely distributed in prokaryotes and the fungal family of eukaryotes.

## 4. PQQ and Plant Growth

There is no evidence that plants produce PQQ; however, PQQ exposure promotes plant growth [43,44,45,46,47,48,49,50,51]. The sources of PQQ for plants are primarily the soil and plant root (rhizome)-associated bacteria. Mechanisms for PQQ—mediated growth stimulation range from increasing soil mineral bioavailability to improving defenses against reactive oxidant species [44,49,50]. For example, rhizobacteria aid in the solubilization of mineral phosphates [43,44,45,46,47,48], whereas *Pseudomonas* mutants, lacking genes responsible for PQQ synthesis, do not promote growth [47]. Treatment of leaf discs with PQQ or wild-type *Pseudomonas fluorescens* results in scavenging of ROS and reductions in hydrogen peroxide. As little as 100 nM of PQQ stimulates growth of *Cucumis sativus* (cucumber) seedlings [44]. PQQ also improves the tolerance of phosphate-solubilizing rhizobacteria to ultraviolet radiation, even when grown under phosphate—free conditions [50]. Improved bacterial tolerance to mitomycin C is also apparent, i.e., fewer DNA strand breaks are observed following PQQ exposure [50]. As a final point, growth promotion may occur by decreasing the harmful effects of plant pathogenic microorganisms. Many rhizobacteria that depend on PQQ for replication produce antibacterial compounds that protect against plant root pathogens such as *Agrobacterium tumefaciens*, which can cause cancerous proliferation of the stem tissue, or *Ralstonia solanacearum*, an aerobic, plant pathogenic bacterium [51].

## 5. PQQ and Insects (*Drosophila Melanogaster* and Nematodes)

### 5.1. Drosophila Melanogaster

*Drosophila melanogaster* and nematodes are often used as models in studies focused on gut microbiology and longevity [52] and surrogates to assess disease vectors at the cell signaling and gene levels [52]. For example, in *D. melanogaster* models of Parkinson’s disease, genetic or pharmacological activation of the *D. melanogaster* homolog of mammalian PGC-1α. rescues the disease phenotype by stimulating *D. melanogaster* PGC-1α. [53]. In turn, silencing *D. melanogaster* PGC-1α results in the expression of Parkinsonian phenotypes [54]. *Drosophila* also depend on commensal bacteria to maintain normal microbiome function. Interestingly, Shin et al. [55] reported that the PQQ-dependent alcohol dehydrogenase (PQQ-ADH) activity of *Acetobacter pomorum*, a commensal acetic acid-producing *Drosophila* bacterium, modulates insulin/insulin-like growth factor (I/IL-GF) signaling. In germ-free *D. melanogaster*, I/IL-GF signaling-related defects are reversed by enhancing host I/IL-GF signaling or supplementing the diet with acetic acid. I/IL-GF signaling influences *D. melanogaster* development, body size, energy metabolism, and intestinal stem cell activity.

### 5.2. Nematodes

In nematodes (*Caenorhabditis elegans*), PQQ exposure enhances nematode antioxidant capacity and extends lifespan by increasing the transcriptional activities of DAF-16/FOXO, SKN-1/NRF2 and SOD-3, factors involved in life extension [56]. DAF-16 is the ortholog of the FOXO family of transcription factors in *C. elegans* and is responsible for activating genes involved in survival (e.g., those associated with telomere extension and oxidative stress). The SKN-1 gene encodes a transcription factor that resembles mammalian NRF-2, which is associated with protection against oxidative damage and inflammation. Finally, Sasakura et al. [57] reported that PQQ activates DUOX protein BLI-3. BLI-3 controls the levels of reactive oxidant species, ensuring the levels are appropriate for optimal growth yet below those that promote inflammation.

## 6. Nutritional Importance in Animal Models and Human Subjects

### 6.1. Murine and Rodents

**Figure 4:**
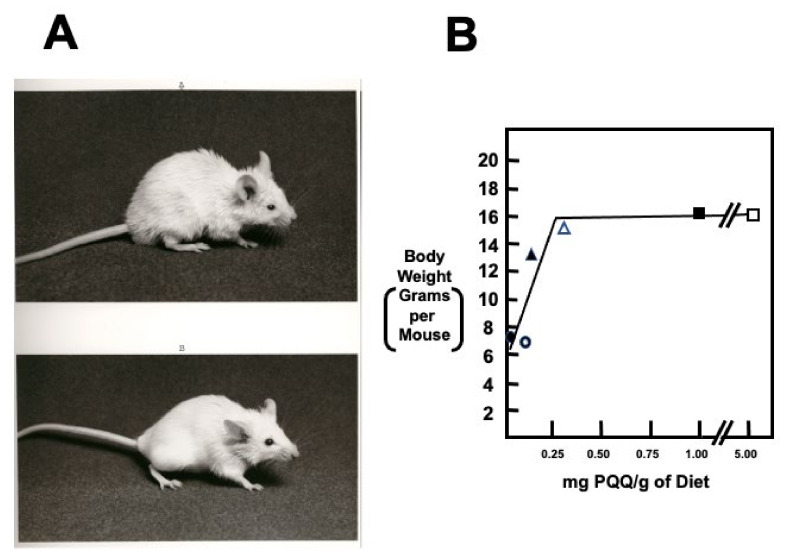
**Growth and appearance of PQQ**-**deprived and** -**supplemented BALB/c mice**. (**A**,**B**) are typical of a PQQ-deprived mouse and control mouse, respectively. Mice fed chemically defined diets devoid of PQQ grow poorly. Severely affected mice have friable skin, hair loss, and a kyphotic appearance. The graph indicates the growth of first-generation mouse pups born from BALB/c dams fed diets containing varying amounts of PQQ. The data suggests that a maternal and subsequent neonatal intake of approximately 300 ng PQQ/g of diet is required for optimal neonatal growth. Values are the means for a minimum of ten 6-week-old mice per. group. Additional details may be found in Steinberg et al. [60,61].

Diet is the apparent source of PQQ in animals given that little, if any, PQQ appears to be synthesized by the intestinal microflora (cf., 3. PQQ’s role in Prokaryotes and Fungi; [58,59]). In nutritional studies, delayed growth and neonatal development are features in mice and rats fed diets devoid of PQQ (Figure 4; [60,61]). The neonatal growth rates for mice [60,61] and rats [62] respond to PQQ in a dose-dependent manner. Optimal neonatal growth is achieved at approximately one μmol of PQQ/kg added to basal diets void of PQQ. Moreover, when PQQ exposure is initiated prior to pregnancy, the mice most deprived of PQQ have fewer litters [61].

Importantly, PQQ derivatives are readily absorbed from diets. For example, Smidt et al. [58] administered a dose of C^14^—PQQ (0.42 μCi/μmol) by gavage to Swiss-Webster mice. Sixty percent of the dose was absorbed, and over 80 percent of the absorbed dose was excreted renally. In addition, most of the C^14^—PQQ in the blood (>95%) was associated with the blood cell fraction rather than plasma.

### 6.2. Pigs

Regarding other animal species, Yin et al. [63] measured growth, intestinal morphology, redox status, and the levels of selected cytokines using weanling pigs. Without added PQQ the average daily weight gain was 336 g/day for the 28-day observational period. At 4.5 mg PQQ/kg diet, the rate of weight gain was −380 g/day. SOD and glutathione peroxidase (GPX) levels were also increased in the duodenum, jejunum, and ileum. In addition, PQQ exposure decreased inflammatory cytokines such as interleukin (IL)-1β, IL-2 and interferon-Y. Likewise, Wang et al. [64] examined the offspring of sows fed diets with or without PQQ supplementation. The diets were fed throughout gestation and lactation. The activity and mRNA expression levels of heme oxygenase 1 (HMOXl), SOD 1 and 2, catalase (CAT), GPX 1 and 4, and glutamate-cysteine ligase (GCL), the first-rate limiting enzyme of glutathione synthesis, were all increased in the intestine of piglets exposed to PQQ. Additional observations reported by Zhang et al. [65] are also complementary to those of Wang et al. [64] and Yin et al. [63].

### 6.3. Chickens

Similarly, PQQ elicits responses in avian species consistent with improvements in redox and immune status. Using a basal diet containing approximately 20 μg PQQ/kg of diet, Samuel et al. [66] reported that the incremental addition of PQQ to 800μg/Kg improved antioxidant defenses, decreased inflammation, and had positive effects on growth in young 220 chickens. Moreover, Zheng et al. [67] reported that PQQ at 10 mg/kg of diet countered the inflammatory effects elicited by lipopolysaccharide (LPS). On a molar basis, PQQ was 10 to 100 times more effective than anti-inflammatory drugs such as acetylsalicylic acid and meclofenamate [68], or biofactors such as melatonin [69], in attenuating the effects of LPS.

### 6.4. Humans

In college—age human subjects, Harris et al. [70] reported that PQQ supplementation (5–10 mg per day) reduced C-reactive protein, interleukin-6 levels, and plasma malonaldehyde levels in plasma. In addition, the ratio of blood lactate to pyruvate and the profile of urinary metabolites (estimated by 1H-nuclear magnetic resonance) were consistent with enhanced mitochondrial oxidation. Likewise, Hwang et al. [71] show daily supplementation with 20 mg PQQ optimizes mitochondrial biogenesis in human subjects. Notably, cognitive function and memory also are improved in human subjects, following PQQ supplementation (10–20 mg per day) [72,73,74].

## 7. PQQ and Its Derivatives in Diets and Supplements

**Figure 5:**
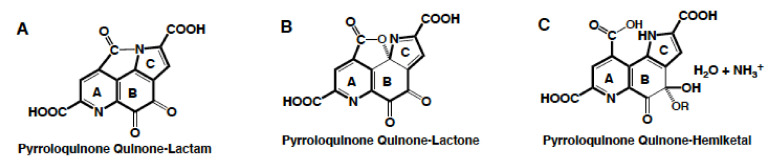
**PQQ derivatives.** PQQ can tautomerize under acidic conditions to form lactones (**B**), lactams (**A**) and hemiketals (**C**). In addition, PQQ readily engages in nucleophilic additions and substitutions.

The assays used for PQQ determination, both spectrophotometric and enzymatic, are sensitive and precise but typically measure only PQQ [75,76,77,78]. Regrettably, precise quantitation of PQQ plus its derivatives is difficult. Using mass spectrometry-based analytical approaches is challenging because of the complex gas-phase fragmentation of PQQ [75]. PQQ-derived ions can arise through acid-catalyzed tautomeric lactonization of PQQ and subsequent oxidation of the PQQ lactone (PQQL) (cf. Figure 5). Decarboxylation of PQQ also occurs easily. Accordingly, the quantitation of total PQQ, including derivatives, is usually qualitative at best. When mass spectrometric analysis has been successfully used to assay PQQ and related derivatives, the levels can be more than an order of magnitude greater than for PQQ alone [75]. For example, in human milk, the concentration of PQQ is estimated to be 20-30 μg/L. Whereas PQQ plus imidazolopyrroloquinoline (IPQ), a derivative easily formed when PQQ reacts with non-branched chain amino acids, is in the range of 140-180 μg/L (~0.5 μM) or 500 to 750 μg/kg of milk solids (~2 μM). PQQ added to aqueous suspensions of semi-purified diet forms derivatives and adducts rapidly, as observed by Steinberg et al. [60,61] and Stites et al. [79]. For example, the half-life for the rate of PQQ adduct formation or disappearance is 45 min at pH 7.0 and ~60 min at pH 2.5 [60].

Nevertheless, as reported in Section 6, strong inferences may be made regarding the need for PQQ. For example, it is possible to produce an apparent PQQ deficiency using experimental diets in animal models. This is novel in that deficiency signs have not been described for other organic biofactors except for those currently viewed conditional vitamins. Moreover, when the effects of PQQ dietary supplements are reported, they are often observed in the low mg/kg/diet or per 1000 kcal range. Except for anthocyanins [80], other biofactors (quercetin, resveratrol, catechins) require intakes of 100–400 mg/kg diet for measurable antioxidant or immune protection-related effects in animal models and humans.

**Figure 6:**
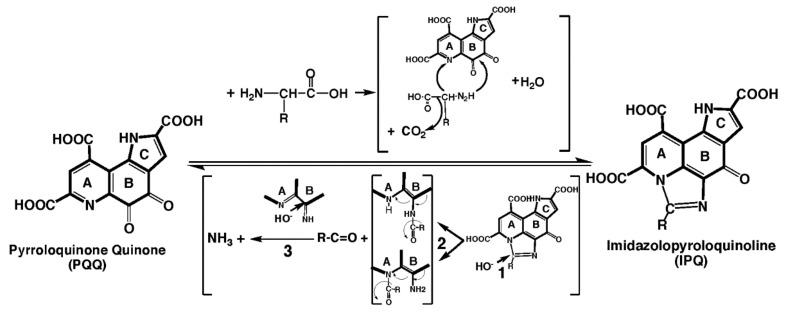
**Imidazolopyrroloquinoline (IPQ).** PQQ reacts with non−branched chain amino acids to form imidazole derivatives (IPQ), with or without retention of the amino acid sidechain (R). The reverse reaction (IPQ → PQQ) most likely results from a base-catalyzed opening of the imidazolium ring as a first step (1). Next, two intermediate forms are possible with the release of the imidazole carbon atom as an aldehyde moiety with or without an attached R group (2). Finally, there is a loss of the imine function that results from nucleophilic addition of OH^−^, isomerization and oxidation (3).

Moreover, IPQ is often as effective as PQQ in bioassays. IPQ is an intermediate when redox cycling is driven by the imidization of amino acids such as L-glycine [81,82,83]; therefore, it can be a potential source of PQQ (Figure 6). Naito et al. [84] measured [^3^H]thymidine incorporation into cultured fibroblast DNA. PQQ significantly enhanced thymidine incorporation in the nM range and IPQ in the μM range. Yamada et al. [85] also compared PQQ and IPQ using human neuroblastoma and hepatocellular carcinoma cell lines, and assays designed to test neuroprotection from 6-hydroxydopamine exposure. Their findings also indicated that IPQ’s biological activity was like that of PQQ. In addition, Tsuchida et al. [86] demonstrated that both PQQ and IPQ are protective in vivo against carbon tetrachloride-induced liver injury in rats.

As a final point, PQQ as a supplement is safe. PQQ meets all the USA-FDA “generally regarded as safe” (GRAS) requirements [87]. Liang et al. [88] have reported that the No-observed-adverse-effect-level (NOAEL) for disodium PQQ is 0.4 g/kg body weight/day in rats for both sexes. The median lethal dose for PQQ in rats is 1.0–2.0 g/kg body weight in males and 0.5–1.0 g/kg body weight in females [89,90].

## 8. Selected Clinical and Organ-Specific Dysfunctions Responsive to PQQ

### 8.1. Kidney and Liver

Chronic kidney disease (CKD) affects almost 15% of the global population [91]. Complications include cardiovascular diseases (CVDs), diabetes, hypertension, anemia, renal disease progression, acute kidney injury, mineral and bone disorders [92], and cognitive decline [93,94,95,96]. The kidney controls body fluid composition by filtering and reabsorbing materials. Reabsorption requires ATP, most of which is generated by mitochondria [97]. The high oxidative activity of mitochondria in the kidney can elevate oxidative stress, resulting in renal dysfunction and CKD progression [98,99]. In both human and animal models, depletion of antioxidant defense activity and increased production of ROS induces inflammatory damage, predominantly targeting renal tubular epithelial cells. The nuclear factor erythroid 2-related factor 2 (NFE2L2/NRF2)—antioxidant responsive element (ARE) prevents the progression of acute kidney injury (AKI) to CKD [100,101] by regulating cellular antioxidant defenses [102]. In multiple studies, a mechanism underlying NFE2L2’s protective role is its action on its target genes, including SOD2 and heme oxygenase (HMOX1), thereby decreasing ROS in the intracellular environment. In experimental CKD models, natural bioactive compounds with kidney protective potential are associated with NFE2L2 activation [103,104]. In this regard, recent work in vivo [105] and in vitro [106] suggests that PQQ appears to target the NFE2L2 pathway. In a mouse model of nephrotoxicity induced with the chemotherapeutic cyclophosphamide (CTX, an immunosuppressive agent), Lin et al. [105] showed PQQ supplementation of CTX-treated mice ameliorates nephrotoxicity by rescuing CTX-mediated 2inhibition of NFE2L2 signaling, characterized by altered expression of NFE2L2 target genes [105].

When treated with high glucose, the HK-1 human proximal tubular epithelial cell line models the progression of diabetes in the kidney. High glucose treatment of HK-1 cells results in elevated ROS and expression of pro-inflammatory genes, with concomitant inhibition of NFE2L2 and its targets. Wang et al. [106] found that PQQ supplementation of HK-1 cells treated with high glucose activates NFE2L2 signaling by increasing NFE2L2 translocation to the nucleus. Furthermore, PQQ mitigates pro-inflammatory signaling both in vivo and in vitro, which Lin et al. showed is regulated through the NLRP3 inflammasome [105].

Factors critical to the programming of neonatal development (e.g., maternal obesity or early exposure to high fat diets) also promote hepatic inflammation. Such processes can lead to nonalcoholic fatty liver disease progression to steatohepatitis in murine models [cf., POO, intestinal barrier functions and the microbiome]. Like the kidney, the expression of NFE2L2 and its target HMOX1, along with NLRP3, is diminished in liver from the offspring of mice fed a Western-style diet and treated with PQQ [107]. In addition, supplementation of PQQ attenuates hepatic inflammation in obese rats [108]. In rats, the amelioration of cadmium- and mercury-induced liver and kidney damage has also been reported following PQQ exposure [109].

### 8.2. Intestinal Barrier Functions and the Microbiome

An intact intestinal barrier is now recognized as an integral regulator of health [110,111,112]. Bacteria and their products are vital contributors to impairment and permeability of the gut barrier, resulting in an increased influx of bacteria, endotoxin, bacterial DNA, and metabolites into the host circulation. Recent reviews show that microbial dysbiosis and impaired barrier function are associated with gastrointestinal disease [113,114,115], neurodegenerative disease [116,117,118,119], autoimmune disease [120,121,122,123,124,125], and an impaired metabolic status in the host manifested by obesity, insulin resistance and cardiovascular complications [126,127,128,129,130,131,132,133,134,135]. In several animal models [63,64,107,136,137], exposure to PQQ increases mRNA expression levels of tight junction proteins and improves jejunal barrier function, suggesting PQQ may act through the gut to affect tissues in the periphery. In this regard, recent evidence has emerged suggesting a short-chain fatty acid, butyrate, is a crucial modulator of barrier function and colonic homeostasis [138,139]. Interestingly, exposure to PQQ leads to increased butyrate levels [137,140], most likely because it is used preferentially as a cofactor for membrane-bound hexose and alcohol dehydrogenases in *E. coli* [141]. As noted previously, membrane-bound hexose and alcohol dehydrogenases are involved in the synthesis of gluconic acid, which is fermented by lactic acid bacteria into lactate and acetate products used by acid-utilizing bacteria to form butyrate [142]. Butyrate exerts a broad spectrum of positive effects, both in the intestine and systemically, including promoting energy expenditure, stimulating peroxisome proliferator-activated receptor coactivator activity, improving insulin resistance, and enhancing mitochondrial function [138,143]. Furthermore, butyrate acts as an inhibitor of histone deacetylase activity [144]; therefore, PQQ-mediated alterations in butyrate levels might result in epigenetic change. Indeed, studies in pigs [137] and rodents [107,136] demonstrated improved barrier function in offspring following supplementation with PQQ. Although studies directly investigating the effects of PQQ on microbiota composition or function are sparse, a current supposition is that some gut bacteria may require PQQ for optimal function.

**Figure 7:**
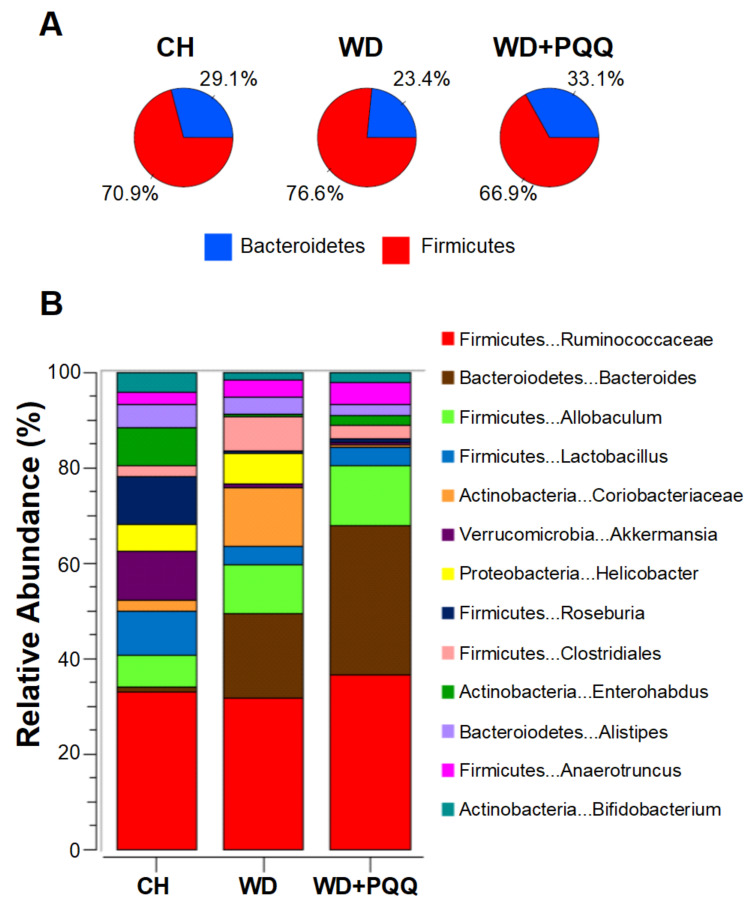
**PQQ**-**associated changes in the gut microbiota of offspring of obese mouse dams.** Obesity in dams was induced by feeding a chow (CH) or Western-style diet (WD), with and without PQQ supplementation. Bacterial compositional differences were measured using 16S sequencing of offspring cecal contents at postnatal day 21 [107]. (**A**) The ratio of *Bacteroidetes* to *Firmicutes* was diminished by WD exposure and rescued by PQQ. (**B**) WD exposure altered relative abundances of the 13 most abundant genera, some of which returned to near control levels in offspring exposed to maternal PQQ.

Using a mouse model of developmental programming of nonalcoholic fatty liver disease, Jonscher and colleagues [107,145] were the first to detect differences in gut bacteria composition in response to PQQ exposure. PQQ supplementation to the dams significantly reversed the diet-induced decrease in *Bacteroidetes* and increases in *Firmicutes* observed in offspring bacterial composition (Figure 7). Genera within both phyla, such as *Bacteroides* and *Allobaculum*, increased in abundance while *Akkermansia*, from the phylum *Verrucomicrobiae*, decreased. Notably, PQQ attenuates the WD-induced bloom of *Clostridiales spp*. In aged rats fed a lard-based high fat diet, *Clostridiales* was the order most affected by diet, and its decrease appeared responsible for gut microbial dysfunction [146]. Whether age or type of fat affects *Clostridiales* abundance remains to be determined, but it is suggestive that this order may be a dynamic, longitudinal biomarker for obesity or lipotoxicity.

### 8.3. Cardiac and Skeletal Muscle Protection

Skeletal and cardiac muscles require a high rate of ATP turnover for efficient contraction. In this regard, rats deficient in PQQ are markedly compromised when subjected to cardiac ischemia/reperfusion injury protocols. For example, Bauerly et al. [147] reported that 20% of rats fed a PQQ deficient diet did not survive ischemia reperfusion, whereas all PQQ supplemented rats survived. These studies were an extension of previous work [148,149,150] in which the cardioprotective effectiveness of PQQ was compared with metoprolol, a beta(l)—selective adrenoceptor antagonist. Rats underwent 30 min of left anterior descending coronary artery occlusion, followed by 2 h of reperfusion. Although both PQQ and metoprolol improved indices of cardiac function, PQQ was superior to metoprolol in protecting mitochondria from ischemia/reperfusion oxidative damage. Whether PQQ supplementation influences cardiac and skeletal muscle function in healthy individuals and animal models remains unclear. For example, Hwang et al. [71] investigated the effects of PQQ supplementation on aerobic exercise performance and indices of mitochondrial biogenesis in men following a six-week endurance training/supplementation program. No differences in aerobic performance were observed; however, there were improvements in peak oxygen consumption and total exercise test duration and recovery. In addition, the PQQ group had a significant increase in PGC—lα protein levels from baseline to post endurance training compared to non-supplemented subjects. In mice, Lui et al. [151] demonstrated that PQQ protects against exercise-induced fatigue and reduced oxidative damage, presumably by improving mitochondrial function.

From a marketing perspective, there are numerous claims made regarding PQQ and exercise performance. The claims are often made without considering that oxygen is the most limiting nutrient influencing exercise performance. Although exposure to compounds with biologic properties like those of PQQ increases mitochondrial content, regular exercise may have similar (or even greater) effects. Body heat regulation is also not often considered. The surface temperature of well-functioning mitochondria is estimated to be 50 °C [152]. Cardiac cell mitochondria appear to decrease ATP production and increase inner-mitochondrial membrane permeability when heat dispersal is compromised, or temperatures are sustained at greater than 40 °C [153]. Thus, it is too soon to conclude that PQQ supplementation can independently improve exercise performance. Indeed, Hwang et al. [71] have recently reported that PQQ supplementation in men undergoing endurance exercise training has little effect on performance apart from an elevation of PGC-lα.

### 8.4. PQQ and Neuroprotection

PQQ is protective in cases and models of brain aging and neurodegeneration, including Parkinson’s disease, stroke, and traumatic brain injury. Perhaps the best of current examples are PQQ’s ability to protect against neuronal agents, such as rotenone [154,155,156,157,158,159,160]. Moreover, the ability of PQQ to protect against neurodegeneration may go beyond mitochondriogenesis, given that PQQ can reduce α—synuclein fibril formation [161]. PQQ also confers resistance to neurocognitive loss in rodent models of stroke and brain injury. Jensen and associates [162] were among the first to use carotid ligation to assess neuroprotective properties following intra peritoneal injection of PQQ. The interruption of blood flow resulted in most of the cortex displaying signs of infarction. Rats without PQQ supplementation had infarctions across −95 percent of cortices compared to −70 percent in rats pretreated with PQQ. In a similar study, Zhang et al. [163] used reversible middle cerebral artery occlusion to simulate interruption of blood circulation to adult rat brains. When PQQ was injected into the jugular vein, either at the same time as occlusion or 3 h after the start of ischemia, markedly improved neurobehavioral scores were observed, along with a reduction in infarct size. Regarding traumatic brain injury, Zhang and associates [155,163,164] assessed spatial memory in rats using the Morris water maze test and demonstrated that PQQ administered intraperitoneally three days before injury improves spatial memory.

In human clinical trials, PQQ was reported to promote cognitive function and improved regional blood flow in older adults [74]. In a randomized placebo-controlled, double-blinded clinical trial, PQQ administered orally (20 mg PQQ/day) to elderly adults resulted in improved cognitive measures. The PQQ dosage was based on a previous animal study showing that PQQ improves spatial memory in rats, as measured by Morris water maze [164].

## 9. Conclusions

As outlined in Table 1, many aspects of PQQ as a biofactor are novel. It is one of the few biofactors for which a nutritional deficiency and a potential nutritional requirement are definable. PQQ acts as an accessory factor for lactate acid and potentially other dehydrogenases in the oxidation of NADH to NAD+. Relationships important to aging, immunity, ROS defense and neurologic integrity involve maintaining optimal NAD+ levels. The effects of PQQ exposure, both in vivo and in vitro, mimic those of cellular NAD+ augmentation [165]. In addition, PQQ is associated with the attenuation of clinically relevant dysfunctions such as ischemia, inflammation, and lipotoxicity. An important observation is that levels of PQQ needed for such effects are usually in the nM to μM range, in contrast to the mM concentrations needed for other popular biofactors. Accordingly, PQQ has strong potential as a therapeutic nutraceutical. A strong case is also evolving, suggesting that PQQ may serve as an essential vitamin-like factor.

## Figures and Tables

**Table 1:** Pyrroloquinoline Quinone: Novel Attributes.

Attribute	Description
PQQ is required for essential physiological functions	PQQ is one of only a few nutritionally important biofactors for which a nutritional deficiency can be defined in multiple species of eukaryotes [9,60,61,66,83]. PQQ has been tentatively identified as a component of interstellar dust [83]. Thus, PQQ may have been present throughout early biological conception and evolution. PQQ is also a plant growth factor [43,44,45,46,47,48,49,50,51], i.e., for animals and humans, there has been constant exposure to PQQ [83].
The apparent need for PQQ is nutritionally attainable without the need for supplementation	PQQ effects are elicited in the nM to μM range of exposure, in contrast to the mM concentrations needed for other biofactors [9,60,61]. PQQ and its derivatives, such as imidazopyrroloquinoline (IPQ) compounds, are found in the milk of mammals in concentrations like vitamins, such as folate, riboflavin and, biotin [12,75]. In rodents, PQQ appears essential for reproduction [60,61,136].
PQQ aids in sustaining cellular NAD^+^ levels and mitochondriogenesis	PQQ acts as an accessory factor for lactate and potentially other dehydrogenases in NADH oxidation to NAD^+^ [18,19]. Accordingly, the effects of PQQ exposure mimic those of cellular NAD^+^ (e.g., sustaining mitochondriogenesis [11,165].
PQQ stimulates sirtuin activity	Sirtuins modulate the activity of factors involved in DNA repair and mitochondriogenesis [23]. PQQ stimulates the expression of proteins in the sirtuin family of enzymes and, by sustaining NAD^+^ levels, optimizes NAD^+^, a sirtuin cofactor and co-substrate [11,165].
Relationships involving PQQ are important to the aging process, immunity, and ROS defense	PQQ exposure increases longevity in models used in the study of aging [53], improves immune responsiveness to cytokines [9,70,145], and acts as a potent redox cycling agent that helps maximize ROS defense [9,12,13,16,107,145].
PQQ is associated with the attenuation of clinically relevant dysfunctions such as ischemia, neurogenic losses, inflammation, and lipotoxicity.	PQQ is neuroprotective and appears to improve memory [74,155,162,163,164]. PQQ protects NMDA mediated receptors in neurological injury. PQQ can reverse hepatic steatosis and has potential as a therapeutic for nonalcoholic fatty liver disease (NAFLD) or nonalcoholic steatohepatitis (NASH) [107,145]. PQQ also influences serum lipid metabolism in ways relevant to protection from heart disease [147]. Moreover, in models of ischemia and reperfusion injury, the cardioprotective effectiveness of PQQ is equivalent to β1-selective adrenoceptor antagonists, such as metoprolol [149]. PQQ alleviates jejunal mucosal inflammatory injury in animal models by inhibiting events associated with NF-κB-related pathways and improving the imbalance of colonic microbiota caused by various agents [9,107,145]. As noted by Naveed et al. [49], inflammation of the gastrointestinal tract has a strong association with ROS genesis. PQQ, as a ROS scavenger, acts as a protectant. PQQ also acts to reduce C-reactive protein and IL-6 levels [61].
PQQ is safe.	The no observed effect level (NOAEL) for PQQ has been determined to be 400 mg/kg bw/day in a sub chronic toxicity study in rats. By applying a safety margin of 100, it can be concluded that doses up to 4 mg/kg BW/day or 240 mg/person/day would be safe in adult humans weighing 60 kg [87,88,89,90].

## Data Availability

Not applicable.
